# Fixed-loop vs. adjustable-loop cortical button devices for femoral fixation in ACL reconstruction – a systematic review and meta-analysis

**DOI:** 10.1186/s40634-022-00544-1

**Published:** 2022-10-21

**Authors:** Simone Birkebæk Elmholt, Torsten Grønbech Nielsen, Martin Lind

**Affiliations:** Department of Orthopaedics, Aarhus University Hospital, Palle Juul-Jensens Boulevard 99, 8200 Aarhus N, Denmark

## Abstract

**Purpose:**

Button implants with either a fixed-loop device (FLD) or adjustable-loop device (ALD) are used frequently in Anterior Cruciate Ligament Reconstruction (ACLR). Since revision ACLR is associated with poorer clinical outcomes, it is important to investigate the difference in risk of revision between FLDs and ALDs. Therefore, this paper aims to systematically assess the risk of revision ACLR between ALDs and FLDs as well as secondary outcomes such as knee stability and patient reported outcomes (PROMs).

**Methods:**

The online databases Embase, Medline (PubMed), and SPORTDiscus were searched, comparing FLDs and ALDs for femoral fixation in patients undergoing primary ACLR with hamstring autografts. Risk of bias was assessed with the ROBINS-I tool for non-randomised studies. Due to heterogeneity a meta-analysis on revision rates were not possible. A random-effect meta-analysis was performed for the secondary outcomes and the quality of evidence was evaluated using the GRADE approach.

**Results:**

Fifteen cohort studies with a total of 2686 patients were included. None of the studies found a clinical difference between ALDs and FLDs in either revision rates, knee stability or PROMS. However, the quality of evidence was graded “very low” due to study designs, risk of bias, and heterogeneity.

**Conclusion:**

Studies of better quality are needed to investigate the risk of revision ACLR between ALDs and FLDs. There was no difference in knee stability and PROMs between the ALDs and FLDs; however, the interpretation of these results is challenging due to low quality of evidence.

**Level of evidence:**

Level III.

## Introduction

Anterior cruciate ligament reconstruction (ACLR) is a commonly performed surgical procedure that aims to re-establish knee stability after an ACL tear. Cortical button devices are frequently used to fixate the ACL graft onto the femur bone with either a fixed-loop device (FLD) or an adjustable-loop-device (ALD) [13]. When using the FLDs, there is the need for an additional drilling depth for button flipping, which results in extra femoral bone loss. Therefore, the ALDs were designed with an adjustable loop allowing for loop re-tensioning after graft insertion and adjustment of the loop’s length [39]. This provides surgical advantages that potentially lead to reduced bone removal [27], reduced femoral tunnel widening [14, 25], and improved graft healing [23]. Biomechanical studies found ALDs to be inferior to FLDs in terms of maximum displacement after cyclic loading, ultimate load to failure, and stiffness [7, 8, 29, 38]. In contrast, clinical studies reported similar knee laxity and patient-reported outcome measures between ALDs and FLDs [1, 5, 9]. These findings have previously been reported in systematic reviews [30, 37].

However, there are no reviews with revision ACLR as primary outcome measure even though revision surgery is the ultimate failure outcome [26]. Furthermore, revision surgery is associated with poorer patient-reported outcomes (PROM) and a higher incidence of cartilage injury with subsequent development of osteoarthritis [24, 42]. Therefore, the rate of revision surgery is an important outcome when evaluating if ALDs are as safe to use in ACLR as FLDs. New clinical studies that include revision rates have been published in recent years [11, 28, 31, 43]. These studies may allow for conducting a meta-analysis that has hitherto been difficult due to heterogeneity between studies.

A systematic review which updates the latest research and include revision surgery as primary outcome measure would contribute to the existing research. Thus, this study aimed to review and perform a meta-analysis of studies that compared revision rates between ALDs and FLDs. Knee laxity and PROMs were included as secondary outcomes. The hypothesis was that the ALDs showed similar revision rates compared with FLDs.

## Materials and methods

The study was performed as a systematic review and meta-analysis in accordance with the Preferred Reporting Items for Systematic Reviews and Meta-analyses (PRISMA) criteria [32].

### Literature search strategy

This systematic review was registered on the Prospero registration site (ID: CRD42021285255). Literature searches were conducted between 30 November and 15 December 2021 in the following electronic databases: Embase (Embase.com), Medline (PubMed host), and SPORTDiscus (EBSCO host). The search was limited to full articles and published studies written in English. The full line search was: “((((“ACL”) OR (“anterior cruciate ligament”)) OR (“Anterior Cruciate Ligament”[Mesh] OR “Anterior Cruciate Ligament Reconstruction”[Mesh] OR “Anterior Cruciate Ligament Injuries”[Mesh])) AND (((“Endobutton”) OR (“Retrobutton”) OR (“XO button”) OR (“Rigidloop”)) OR ((“fixed” OR “fixation”) AND (loop OR button OR “length”)))) AND ((“Zipploop” OR “Tight Rope” OR “Rigidloop” OR “UltraButton”) OR ((“Adjustable” OR “variable”) AND (loop OR button OR length))).”

The Population, Intervention, Comparison, and Study (PICOS) principles guided the search strategy [10]. Databases were searched for studies that met the following criteria: investigating revision surgery, knee laxity, or PROMs for patients receiving ACLR performed with hamstring tendon autografts and comparing adjustable-loop devices to fixed-loop devices for femoral graft fixation.

The reference list of included studies and systematic reviews, conducted on the same area known by the authors, were searched for additional studies.

### Selection process

**Fig. 1:**
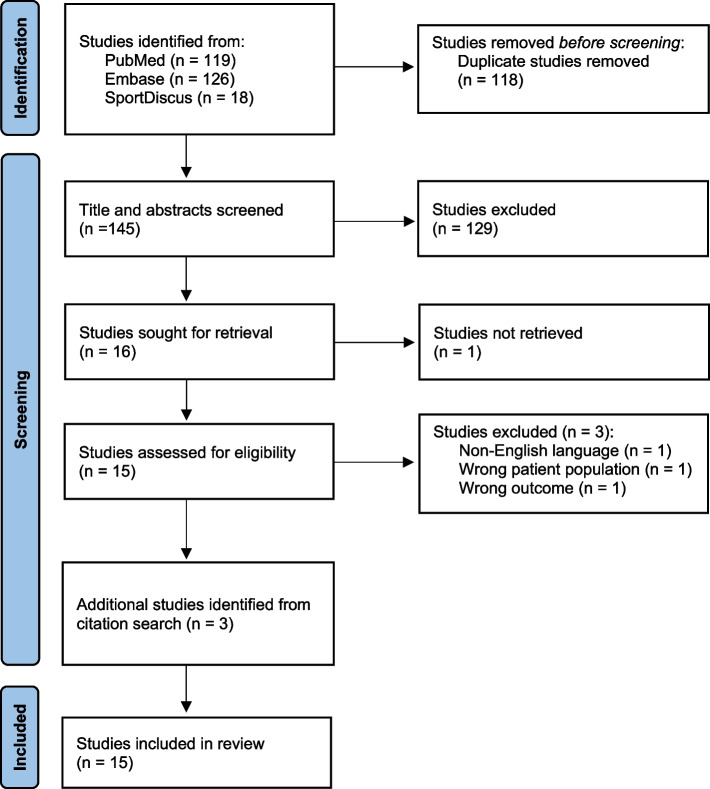
Flow chart of study selection and inclusion process

The selection process was conducted using the online software Covidence. Two authors (SE) and (TN) independently conducted title and abstract screening and any discrepancies were resolved through discussions. Full text screening of included studies was carried out in the same way. The reference list from included studies and from existing reviews [30, 37] on the same topic known by the authors were searched for additional eligible studies by a single assessor (SE). Studies identified from the reference lists were screened in full text by both assessors to reach a final agreement. Studies were considered eligible is they met the PICOS criteria. The identification and screening process are outlined in Fig. 1.

### Data extraction

Data extractions were performed using a predefined data extraction form created on the online software, Covidence. The following data was extracted through the extraction form: type of study, fixation-device fabricant, tibial fixation, number of included patients, distribution of sex and age, follow-up time, and outcome data. The rate of revision surgery was the primary outcome and was defined as a second ACLR performed on the same knee as the primary ACLR. Secondary outcomes included anterior knee laxity, measured with arthrometers (KT-1000 or Rolimeter) as the side-to-side difference (SSD) in mm between the reconstructed and healthy knee, and PROMs as measured by the International Knee Documentation Committee (IKDC) [21] and Lysholm score [6]. Two reviewers (SE) and (TN) conducted data extractions independently and discrepancies were discussed to reach a consensus.

### Quality assessment

The Cochrane Risk of Bias In Non-Randomized Studies of Interventions (ROBINS-I) [40] was used to evaluate the risk of bias in each of the included studies. A single assessor (SE) completed the bias assessment and made an overall risk of bias assessment for each article. The results were afterwards presented to the co-authors. If a decision was unclear, the authors discussed it to reach an agreement.

The ROBINS-I contains the following seven domains of bias: due to confounding, in selecting participants, in classifying interventions, due to deviations from intended interventions, due to missing data, in measuring outcomes, and in selecting the reported result. Bias was assessed separately for each of the three outcomes: revision surgery, knee laxity, and PROMs. The important factors included in the confounding domain for each outcome were chosen after a discussion between the authors. Important confounders of revision rates and knee laxity included the use of more than one tibial fixation device, the different surgical techniques used (i.e. anteromedial, transtibial, outside-in), age, and surgery on other ligaments (i.e. posterior cruciate ligament, medial collateral ligament, or lateral collateral ligament). Important confounders for the PROMs were age and knee comorbidities (osteoarthritis/cartilage damage, meniscus injury, other ligament injuries). Rehabilitation was considered an important co-intervention for all outcomes. If the study included different rehabilitation programmes or did not report on the rehabilitation protocol, it was considered as having a moderate risk of bias in the domain of deviations from the intended intervention. For the missing data domain, it was decided that missing data for 10–19% of the participants resulted in a moderate risk of bias and missing data for > 20% of the participants resulted in a serious risk of bias. The overall risk of bias was determined by the worst degree of bias assessed across all bias domains [40].

The Grading of Recommendations Assessment, Development, and Evaluation (GRADE) was used to evaluate the quality of evidence for each meta-analysis [17]. A single assessor (SE) conducted the GRADE evaluation, after all authors approved it.

### Data analysis

The rates of revision surgery are reported as categorical data. The risks ratio of revision surgery is presented by an Odds ratio (OR) with a 95% confidence interval (CI). If the included studies did not report the OR, it was calculated by using the proportions stated in the study. A meta-analysis of the pooled effect size of revision rates was not estimated due to high heterogeneity between the included studies. Knee laxity by SSD and PROMs are reported as continuous data by a mean difference (MD). Random effect models were used to estimate the pooled effect of the MD for the SSD and PROMs with a 95% CI at the 2-years follow-up. Heterogeneity due to variations across the studies were assessed using the I^2^ test [19]. Publication bias assessed using funnel plots were not possible due to the low number of studies included. The statistical analyses were conducted using Stata version 17 (StataCorp, College Station, Texas).

## Results

A total of 15 studies were included in this systematic review [1, 3–5, 9, 11, 14, 25, 28, 31, 33, 34, 36, 41, 43]. All studies were screened in full text. Four studies did not meet eligibility criteria: one could not be retrieved in full text [20], one was not written in English [12], one was conducted with several graft types [44], and presented other clinical outcomes than those included by this review [45]. Three additional studies were identified from the reference list of included studies [4, 14, 33]. Key characteristics for each of the included studies are presented in Table 1.

**Table 1:** Study characteristics

Study (year)	Study design	FLD device	ALD device	Tibial fixation	Patients			Age (mean, SD)		Sex, Males (n, %)	
					**Total**	**FLD**	**ALD**	**FLD**	**ALD**	**FLD**	**ADL**
Firat et al. (2014) [14]	Retrospective	Endobutton	ToggleLoc with ZipLoop	Bioderadable screw	78	46	32	28.4 (4.2)	27.7 (4.2)	35 (76)	25 (78)
Boyle et al. (2015) [5]	Retrospective	Retrobutton	TightRope	Staple and interference screw	188	115	73	26.1 (11.0)	25.8 (11.7)	64 (56)	50 (68)
Lanzetti et al. (2016) [25]	Prospective	Endobutton	TightRope	Bioderadable screw	44	22	22	26.1 (3.4)	25.2 (3.6)	16 (73)	17 (77)
Choi et al. (2017) [9]	Retrospective	Endobutton	TightRope	Bioderadable screw	117	67	50	29.9 (11.5)	28.2 (11.6)	55 (82)	41 (82)
Sundararajan (2018) [41]	Retrospective	Endobutton	TightRope	Bioderadable screw	98	44	54	37^b^^,d^	33^b^^,d^	38 (86)	41 (76)
Pokharel et al. (2018) [33]	Prospective	Endobutton	TightRope	Bioderadable screw	60	30	30	33 (18.2)	31 (20)	26 (87)	24 (80)
Asmussen et al. (2018) [4]	Retrospective Register-based	Endobutton	ToggleLoc with ZipLoop	Various types	1654	1538	116	26.1^d^	25.9^d^	860 (56)	71 (61)
Ranjan et al. (2018) [34]	Prospective	Endobutton	TightRope	Bioderadable screw	102	52	50	28.7 (9.5)	30.4 (7.9)	41 (79)	40 (80)
Ahn et al. (2018) [2]	Retrospective	Retrobutton	TightRope	Bioderadable screw	29	22	17	30.7 (10.6)^c^	32.6 (10.8)^c^	39 (81)^c^	51 (84)^c^
Sheth et al. (2019) [36]	Prospective	Endobutton	TightRope	Bioderadable screw	62	31	31	30.1 (8.9)	30.8 (6.9)	25 (81)	24 (77)
Ahn et al. (2019) [1]	Prospective	Endobutton	TightRope	Bioderadable screw	79	41	38	32 (8.2)	31.2 (10.8)	31 (76)	29 (76)
Uribe et al. (2020) [43]	Prospective	Endobutton	TightRope	Interference screw^a^	27	13	24	33.9 (11.2)	31.3 (11.7)	6 (46)	11 (46)
Mohamed et al. (2020) [28]	Prospective	Not described	Not described	Bioderadable screw	60	30	30	27.2 (3.3)	26.9 (2.5)	30 (100)	30 (100)
Ono et al. (2021) [31]	Prospective	Endobutton	TightRope	TightRope	28	13	15	25.2 (9.6)	25.7 (8.4)	6 (46)	8 (53)
Djordjevíc et al. (2021) [11]	Prospective	VersiTomic G-Lok	TightRope	Bioderadable screw	60	30	30	27.9 (6.9)	26.9 (6.4)	26 (87)	27 (90)

^a^Three patients received an ALD for tibial fixation

^b^Described as “average age” in the study

^c^Patient characteristics measured before lost to follow-up

^d^Standard deviations not available

### Revision surgery

Three studies [4, 5, 43] included revision surgery in their outcome and two studies [28, 34] reported on the number of patients undergoing revision surgery during the study period. Only the study by Asmussen et al. [4] analysed the rate of revision surgery with an OR and found that ALDs had 0.51 (95% CI: 0.24–1.13) lower odds of having a revision surgery compared to FLDs. However, this was not statistically significant [4]. Boyle et al. [5] and Ranjan et al. [34] did not present an OR for the risk of having a revision surgery in the ALD group compared to the FLD group. However, this study calculated an OR based on the proportions of revision surgery reported by both Boyle et al. [5] and Ranjan et al. [34]. The results are presented in Table 2. Mohamed et al. [28] found only one.

**Table 2:** Revision rates presented as proportions and odds ratio

Study	ALD-group (n events/n total (%))	FLD-group (n events/n total (%))	OR (95% CI)	Follow-up time	Classification of revision
Boyle 2015 [5]	7/73 (10)	13/115 (11)	0.83 (0.32–2.19)	2 years	Revision surgery due to graft failure^a^
Ranjan 2018 [34]	1/50 (2)	2/52 (3.8)	0.51 (0.04–5.56)	2 years	Failure due to reinjury
Asmussen 2018 [4]	7/116 (1.9)	102/1538 (3.6)	0.52 (0.24–1.13)	FLD = 929 days^b^ ALD = 743 days^b^	Revision surgery
Mohamed 2020 [28]	2/30 (6.7)	0/30	-	1 year	Revision surgery due to unsatisfactory results^c^
Uribe 2020 [43]	0/24 (0)	0/13 (0)	-	2 years	Revision surgery

^a^Failure defined as either a grade 2 + Lachmann, a positive pivot shift or an SSD greater than five millimetres

^b^Mean follow-up

^c^Patients experiences giving away, locking and difficulties with climbing stairs

case of revision surgery and this was in the ALD group. Uribe et al. [43] reported no cases of revision surgery in either of the groups.

### Knee laxity

The SSD are reported at the 6 months follow-up in three studies [5, 34, 43], at the 1-year follow-up in three studies [4, 25, 43], and at the 2-years follow-up in five studies [5, 9, 11, 14, 34]. There was no difference in the mean SSD between the ALDs and FLDs in either of the studies Table 3.

**Table 3:** KT-1000 side-to-side difference measured at 6 months, 1 year and 2 years follow-up

Study	6 months*		1 year*		2 years*	
	**ALD**	**FLD**	**ALD**	**FLD**	**ALD**	**FLD**
Boyle et al. [5]	1.51 (1.4)	1.79 (1.5)	1.44 (1.4)	1.64 (1.4)	1.14 (1.5)	1.07 (1.1)
Ranjan et al. [34]	0.4 (1.26)	0.6 (1)	-	-	0.16 (1.33)	0.12 (0.92)
Uribe et al. [43]	1.7 (2.4)	1.8 (2.6)	-	-	-	-
Asmussen et al. [4]	-	-	0.83 (1.7)	1.25 (1.9)	-	-
Lanzetti et al. [25]	-	-	2.1 (1.2)	2.3 (1)	-	-
Firat et al. [14]	-	-	-	-	2.5 (0.8)	2.3 (1.0)
Choi et al. [9]	-	-	-	-	1.2 (2.3)	1.5 (1.8)
Djordjevíc et al. [11]	-	-	-	-	1.10 (0.89)	1.17 (0.78)

Data are presented as mean $\pm$ SD

^*^All results were non-significant with a *p*-value > 0.05

**Fig. 2:**
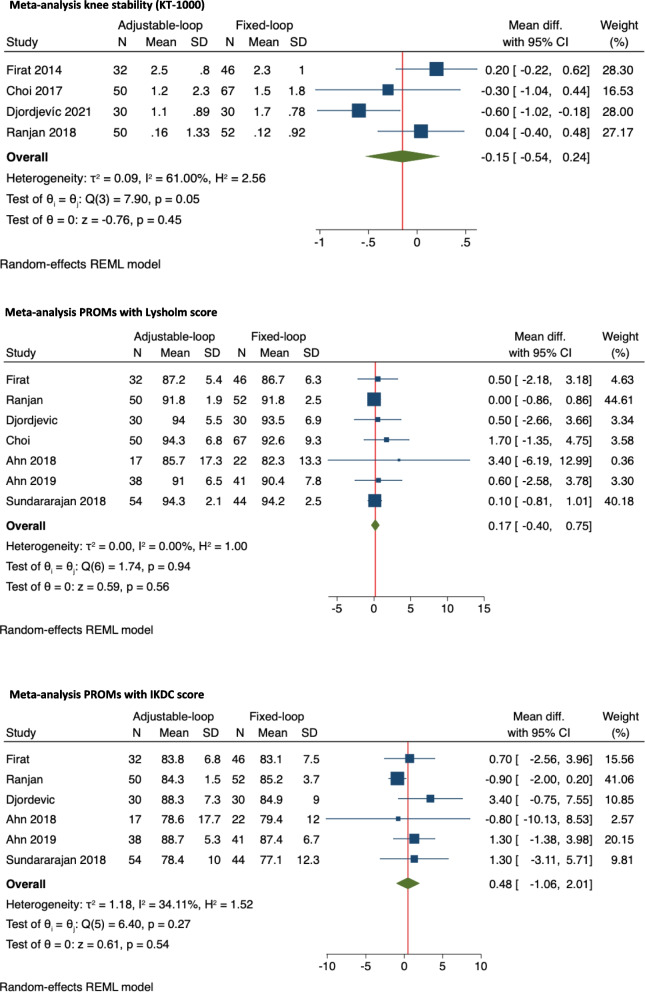
Meta-analysis of knee laxity and PROMs with the Lysholm and IKDC score

The meta-analysis revealed an overall MD in the SSD at the 2-years follow-up of -0.15 mm (95% CI: -0.54—0.24) lower in the ALD group compared to the FLD group (Fig. 2). The I^2^ test revealed a high heterogeneity of 61% across the studies.

### Patient-reported outcomes

Five studies reported the PROMs with the Lysholm score at the 1-year follow-up [25, 28, 31, 33, 36] and seven studies reported them at the 2-years follow-up [1, 2, 9, 11, 14, 34, 41] (Table 4). The meta-analysis for the 2-years Lysholm scores revealed an overall MD in the Lysholm score of 0.17 points (95% CI: -040 – 0.75) higher for the ALD group compared to the FLD group (Fig. 2). Three studies reported the PROMs with the IKDC score at the 1-year follow-up [25, 33, 36] and six studies reported it at the 2-years follow-up [1, 2, 11, 14, 34, 41] as presented in Table 4.

**Table 4:** Lysholm and IKDC scores at 1 and 2-years follow-up

Follow-up	Study (year)	Lysholm Score				IKDC Score			
		**ALD group**		**FLD group**		**ALD group**		**FLD group**	
		**Pre-op**	**Post-op**	**Pre-op**	**Post-op**	**Pre-op**	**Post-op**	**Pre-op**	**Post-op**
**1 year**	Lanzetti (2016) [25]^a^	-	93.2	-	92.8	-	90.4	-	89.5
	Pokharel (2018) [33]	56.5 (7.1)	94.7 (3.7)	56.63 (6.7)	93.97 (4.1)	46.57 (6.5)	83.98 (4.1)	46.16 (6.1)	82.52 (4.2)
	Sheth (2019) [36]	35.5 (5.2)	94.3 (2.1)	34.5 (5.4)	94.2 (2.5)	33.3 (3.4)	92.2 (2.1)	33.5 (2.8)	92.0 (1.9)
	Mohamed (2020) [28]	55.2 (9.2)	93.0 (9.0)	57.5 (7.4)	95.0 (6.4)	-	-	-	-
	Ono (2021) [31]	71.6 (19.3)	86.7 (13.3)	69.7 (22.4)	94.9 (8.3)	-	-	-	-
**2 years**	Firat (2014) [14]	-	87.2 (5.4)	-	86.7 (6.3)	-	83.8 (6.8)	-	83.1 (7.5)
	Choi (2017) [9]	58.1 (16.2)	94.3 (6.8)	58.3 (16.6)	92.6 (9.3)	-		-	
	Sundararajan (2018) [41]	-	87.3 (4.0)	-	87.3 (4.4)	-	78.4 (10.0	-	77.1 (12.3)
	Ranjan (2018) [34]	52 (7.1)	91.8 (1.94)	53.2 (8.6)	91.8 (2.45)	38.5 (4.9)	84.3 (1.52)	37 (6.6)	85.2 (3.66)
	Ahn (2018) [2]	52.2 (23.0)	85.7 (17.3)	63.0 (21.0)	82.3 (13.3)	51.2 (24.7)	78.6 (17.7)	53.2 (19.5)	79.43 (12.0)
	Ahn (2019) [1]	61.2 (8.6)	91.0 (6.5)	55.5 (6.8)	-	39.6 (6.9)	88.7 (5.3)	43.3 (8.5)	87.4 (6.7)
	Djordjevíc (2021) [11]	-	94.0 (5.5)	90.4 (7.8)	93.5 (6.9)	-	88.3 (7.3)	-	84.9 (9.0)

^a^Standard deviation (SD) not reported

The meta-analysis for the 2-years IKDC scores revealed an overall MD in the IKDC score of 0.48 points (95% CI: -1.06 – 2.01) higher for the ALD group compared to the FLD group (Fig. 2).

### Quality assessment

**Fig. 3:**
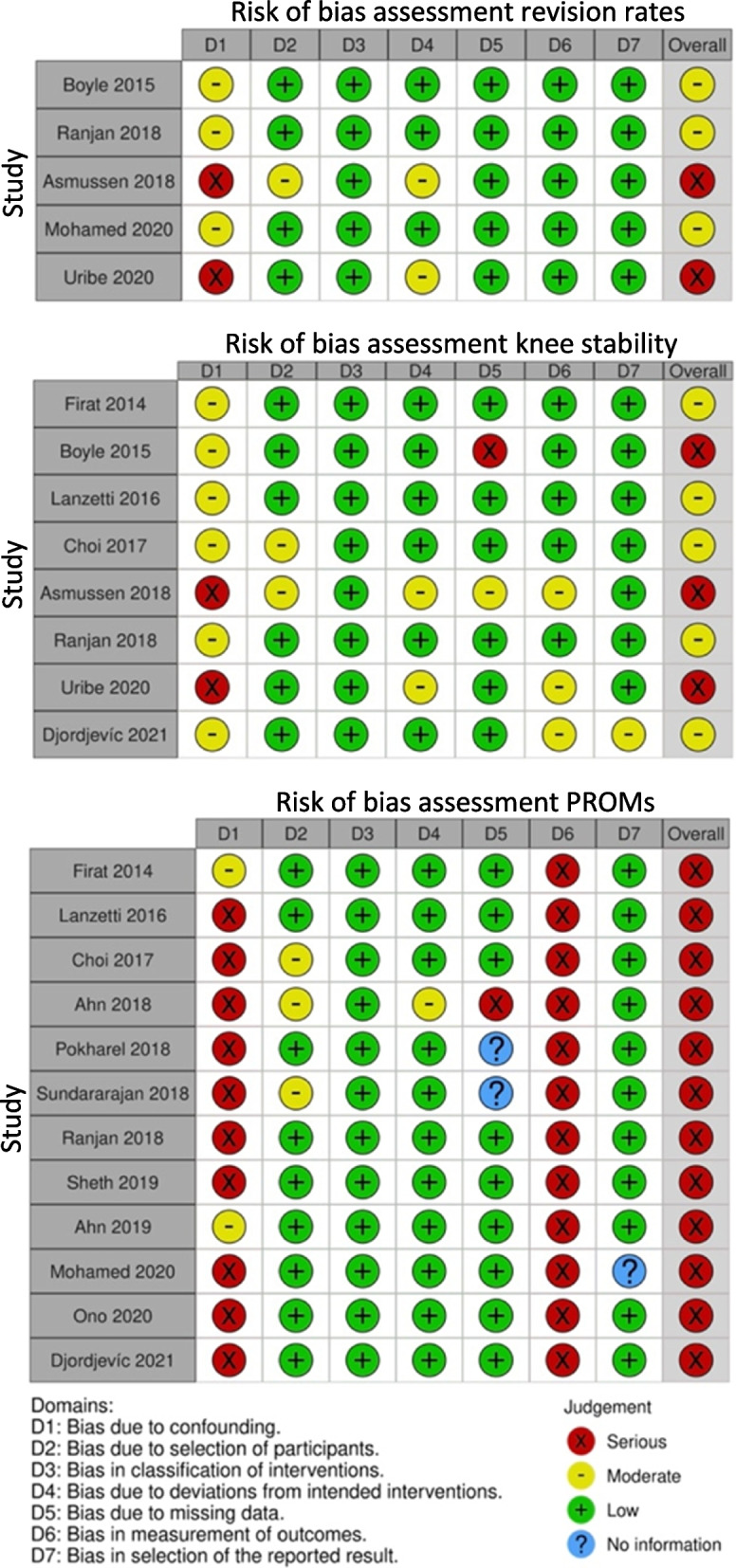
Individual risk of bias assessment for all included outcomes

The overall risk of bias assessment showed that the included studies ranged from an overall moderate risk of bias to an overall serious risk of bias depending on the outcome measure (Fig. 3).

For the bias assessment of revision rates and knee laxity, the overall serious risk of bias judgement was mainly due to confounding. Two studies were judged as having a serious risk of bias due to confounders. One used several tibial fixation methods [4] and the other used two different surgical techniques [43]. Most of the studies had a low or moderate risk of bias regarding selection of participants, deviations from intervention, measurement of outcomes, and selection in reported results. For the knee laxity outcome, one study had a serious risk of bias due to missing data [5].

In the bias assessment of the PROMs, the overall risk of bias was serious for all studies. This was mainly because of bias due to confounders and in measurement of the outcome since this was a subjective measure and thereby could be influenced by knowledge of intervention. Most of the studies had a low or moderate risk of bias regarding selection of participants, deviations from intervention, and selection in reported results. One study had a serious risk of bias due to missing data [2]. The risk of bias in classifying interventions was at a low risk for all studies in all outcomes.

The GRADE evaluation demonstrated a “very low” quality of evidence for each of the three meta-analyses (Table 5).

**Table 5:** Summary of findings table

Adjustable-loop devices compared to Fixed-loop devices for femoral graft fixation in Anterior Cruciate Ligament Reconstruction				
Patient or population: femoral graft fixation in Anterior Cruciate Ligament Reconstruction Setting: Patients undergoing ACL reconstruction Intervention: Adjustable-loop devices Comparison: Fixed-loop devices				
Outcomes	**Anticipated absolute effects**^**a**^ (95% CI)		№ of participants (studies)	Certainty of the evidence (GRADE)
	**Means in Fixed-loop devices**	**MD with Adjustable-loop devices**		
Knee Laxity Assessed with: KT-1000 Scale from: 0 mm to 10 mm Follow-up: 2 years	The mean knee Laxity was **1.4** mm^b^	MD **0.15 mm lower** [0.54—0.24]	357 (4 observational studies)	⨁◯◯◯^c,d^ Very low
Patient Reported Knee Function (PROMS) Assessed with: Lysholm score Scale from: 0 to 100 Follow-up: 2 years	The mean patient Reported Knee Function was **89.5** Points^b^	MD **0.22 Points higher** [0.52—0.97]	475 (6 observational studies)	⨁◯◯◯^c,e^ Very low
Patient Reported Knee Functions (PROMS) assessed with: IKDC score Scale from: 0 to 100 Follow-up: 2 years	The mean patient Reported Knee Functions was **84** Points^b^	MD **0.43 Points higher** [1.25—2.11]	358 (5 observational studies)	⨁◯◯◯^c,e^ Very low

GRADE Working Group grades of evidence

High certainty: we are very confident that the true effect lies close to that of the estimate of the effect

Moderate certainty: we are moderately confident in the effect estimate: the true effect is likely to be close to the estimate of the effect, but there is a possibility that it is substantially different

Low certainty: our confidence in the effect estimate is limited: the true effect may be substantially different from the estimate of the effect

Very low certainty: we have very little confidence in the effect estimate: the true effect is likely to be substantially different from the estimate of effect

*CI* Confidence interval, *MD* Mean difference

^a^The risk in the intervention group (and its 95% confidence interval) is based on the assumed risk in the comparison group and the relative effect of the intervention (and its 95% CI)

^b^Fixed-loop group mean laxity and PROMS scores are calculated from pooled estimates

^c^All outcomes are downgraded to by two due to study design: observational studies

^d^Serious inconsistency due to high heterogeneity (I^2^ = 61%) (*p* = 0.05)

^e^Serious risk of bias due to confounding and measurement of outcomes (subjective reported outcomes)

All outcomes were initially downgraded to “low” quality of evidence because of the study design (i.e. observational studies). The meta-analysis of knee laxity was further downgraded to “very low” due to high heterogeneity (I^2^ = 61%). The two meta-analyses of PROMs by the Lysholm score and the IKDC score had a serious risk of bias and was thus downgraded further to “very low”.

## Discussion

The most important finding of this systematic review and meta-analysis was that there was no difference in revision surgery rates comparing ALDs to FLDs for femoral graft fixation using hamstring tendon autografts in ACLR. Furthermore, this systematic review found that the overall risk of bias assessment ranged from moderate to serious and that the quality of evidence in the meta-analyses was “very-low”.

Five studies reported the rate of revision surgery for ALDs and FLDs. Asmussen et al. [4] included the largest patient population in their registry-based cohort study and was the only study that compared the rate of revision surgery between ALDs and FLDs using statistics. They found that ALDs had a lower risk of revision surgery compared to FLDs. This result was not statistically significant, and the study had a serious overall risk of bias. Uribe et al. [43] was the only study that reported no cases of revision surgery; however, their study included the fewest number of patients, which may explain this finding. None of the included studies specified rehabilitation protocols and return-to-sport criteria. Two studies [5, 28] reported that patients returned to sports from 6 months postoperatively and the remaining three studies [4, 34, 43] did not report whether the patients returned to sports. The lack of information in the studies on rehabilitation protocols and return-to-sport criteria is of importance since higher sport activity levels and an early return to sports before meeting the appropriate criteria are associated with a greater risk of reinjury and a possible revision surgery [15, 18]. A meta-analysis was not performed due to heterogeneity of the study design i.e. surgical technique for tunnel drilling, differences in tibial fixation methods, number of patients, and different follow-up time. To reduce bias, studies that only performed ACLR with hamstring tendon autografts were included, since allografts have demonstrated a greater risk of graft failure compared to autografts [16, 22].

Secondary, this systematic review found no difference between ALDs and FLDs in knee laxity and PROMs. The results from the meta-analysis of knee laxity showed that there was no significant difference between ALDs and FLDs in the SSD in anterior knee laxity when measured with the KT-1000 arthrometer. However, due to study design and a large heterogeneity from the I^2^ test, the GRADE evaluation demonstrated a “very low” quality of evidence. The large heterogeneity may be partially explained by differences in the force applied during the KT-1000 measurements. The studies by Choi et al. [9] and Firat et al. [14] stated that the KT-1000 measurements were performed at maximal force as opposed to the studies by Ranjan et al. [34] and Djordjevíc et al. [11], who did not specify the force applied. The inter-reader reliability with the KT-1000 arthrometer was found poor by Runer et al. [35], who stated that this could partially be explained by differences in the force that the assessors applied. The meta-analysis conducted on PROMs showed that ALDs did not improve the PROMs compared to the FLDs when using either the IKDC or the Lysholm score. The GRADE assessment graded the result as a “very low” quality of evidence due to study design and serious risk of bias in the confounding and measurement of outcome domains. Since PROMs are subjective, the measures are considered to run a high risk of bias by the ROBINS-I tool.

To the authors’ knowledge, this review is the first to provide a schematic overview and to update the knowledge on the differences in revision rates between ALDs and FLDs from the latest research. Also, this review is the first to provide a thorough bias assessment with the ROBINS-I [40] bias assessment tool, specifically developed to assess bias in non-randomized studies. Furthermore, this systematic review provides a detailed meta-analyses of knee laxity and PROMs and is the first to evaluate these results using the recognized GRADE approach [17].

The primary aim of this systematic review was to use a meta-analysis to evaluate the risk of revision surgery between ALDs and FLDs. However, this was not possible due to the low number of studies evaluating revision surgery rates and high heterogeneity between the studies. Furthermore, none of the studies presented a sample size calculation based on detecting a difference in revision rates, which raises concerns about the statistical power of these studies. Studies with a powered sample size and a longer follow-up period would contribute to existing research. Another limitation is that all studies included in this systematic review and meta-analysis were cohort studies, thereby impacting the quality of evidence evaluated by GRADE. Following the GRADE approach, it is recommended to grade observational studies as “low” because of their limitations compared to randomised controlled trials.

Despite the low quality of evidence, the results of this systematic review and meta-analysis are in accordance with the findings of previous systematic reviews [30, 37]. Biomechanical studies have previously raised concerns about the use of ALDs due to elongation. However, based on the results from this systematic review and meta-analysis, ALD usage as femoral fixation during ACL reconstruction is not associated with greater knee laxity and higher revision rates than FLD usage.

This systematic review and meta-analysis provide clinicians with a detailed and schematic summary on clinical outcomes between ALDs and FLDs. Furthermore, it indicates that further research on revision rates of better quality could benefit the existing knowledge.

## Conclusion

This systematic review found that there was no difference in revision rates between ALDs and FLDs in either of the included studies. Furthermore, the meta-analysis showed no differences regarding knee laxity and PROMs. These data suggest that both types of loop devices are safe to use in ACLR, supporting the existing research. However, the available clinical studies’ quality is low and shows serious bias risk.

## Abbreviations

ACLR: Anterior cruciate ligament reconstruction
ALD: Adjustable-loop device
FLD: Fixed-loop device
PROM: Patient-reported outcome measures
PRISMA: Preferred Reporting Items for Systematic Reviews and Meta-analyses
PICOS: Population, Intervention, Comparison, and Study
SSD: Side-to-side difference
IKDC: International Knee Documentation Committee
ROBINS-I: Risk of Bias In Non-Randomised Studies – of Interventions
GRADE: Grading of Recommendations Assessment, Development, and Evaluation
CI: Confidence interval
OR: Odds ratio
MD: Mean difference
