# Correction: Fixed-loop vs. adjustable-loop cortical button devices for femoral fixation in ACL reconstruction – a systematic review and meta-analysis

**DOI:** 10.1186/s40634-022-00553-0

**Published:** 2022-11-26

**Authors:** Simone Birkebæk Elmholt, Torsten Grønbech Nielsen, Martin Lind

**Affiliations:** Department of Orthopaedics, Aarhus University Hospital, Palle Juul-Jensens Boulevard 99, 8200 Aarhus N, Denmark

**Correction: J Exp Ortop 9, 106 (2022)**

10.1186/s40634-022-00544-1

This erratum adds an abstract to the article Fixed-loop vs. adjustable-loop cortical button devices for femoral fixation in ACL reconstruction – a systematic review and meta-analysis [1]. The abstract was missing from the article version published 21 Oct 2022.

**Abstract**

**Purpose**

Button implants with either a fixed-loop device (FLD) or adjustable-loop device (ALD) are used frequently in Anterior Cruciate Ligament Reconstruction (ACLR). Since revision ACLR is associated with poorer clinical outcomes, it is important to investigate the difference in risk of revision between FLDs and ALDs. Therefore, this paper aims to systematically assess the risk of revision ACLR between ALDs and FLDs as well as secondary outcomes such as knee stability and patient reported outcomes (PROMs).

**Methods**

The online databases Embase, Medline (PubMed), and SPORTDiscus were searched, comparing FLDs and ALDs for femoral fixation in patients undergoing primary ACLR with hamstring autografts. Risk of bias was assessed with the ROBINS-I tool for non-randomised studies. Due to heterogeneity a meta-analysis on revision rates were not possible. A random-effect meta-analysis was performed for the secondary outcomes and the quality of evidence was evaluated using the GRADE approach.

**Results**

Fifteen cohort studies with a total of 2686 patients were included. None of the studies found a clinical difference between ALDs and FLDs in either revision rates, knee stability or PROMS. However, the quality of evidence was graded “very low” due to study designs, risk of bias, and heterogeneity.

**Conclusions**

Studies of better quality are needed to investigate the risk of revision ACLR between ALDs and FLDs. There was no difference in knee stability and PROMs between the ALDs and FLDs; however, the interpretation of these results is challenging due to low quality of evidence.

**Level of evidence**

Level III

The original article [1] has been corrected.
